# Clinical Features, Phenotypic Markers and Outcomes of Diffuse Large B-Cell Lymphoma between HIV-Infected and HIV-Uninfected Chinese Patients

**DOI:** 10.3390/cancers14215380

**Published:** 2022-10-31

**Authors:** Minghan Zhou, Jinlin Cheng, Handan Zhao, Min Yang, Wenjuan Yu, Jiaying Qin, Guanjing Lang, Ran Tao, Qing Cao, Ying Huang, Biao Zhu, Lijun Xu

**Affiliations:** 1 Department of Infectious Diseases, The First Affiliated Hospital, College of Medicine, Zhejiang University, Hangzhou 310003, China; 2 National Clinical Research Center for Infectious Diseases, The First Affiliated Hospital, College of Medicine, Zhejiang University, Hangzhou 310003, China; 3 The State Key Laboratory for Diagnosis and Treatment of Infectious Diseases, The First Affiliated Hospital, College of Medicine, Zhejiang University, Hangzhou 310003, China; 4 Department of Hematology, The First Affiliated Hospital, College of Medicine, Zhejiang University, Hangzhou 310003, China

**Keywords:** lymphoma, HIV, phenotypic markers, prognosis

## Abstract

**Simple Summary:**

Diffuse large B-cell lymphoma (DLBCL) frequently occurs in HIV-infected patients. However, the effect of HIV infection on the outcome of the DLBCL population remains controversial. The aim of the present retrospective study was to compare clinical features, phenotypic markers and outcomes of BLBCL between HIV-infected and HIV-uninfected Chinese patients. Our study indicated HIV-infected DLBCL patients displayed high EBER expression but low CD20 and CD79a expression on histopathology. The overall response rate at end of chemotherapy and 1-year overall survival (OS) were low in HIV-infected patients, which may be associated with the higher incidence of leukopenia, neutropenia, thrombocytopenia and hypoalbuminemia, as well as high involvement of the central nervous system (CNS), gastrointestinal tract and bone marrow. Hypoalbuminemia and CNS involvement were independent risk factors for 1-year OS. HIV-infected DLBCL patients without CNS involvement had a favorable outcome if rituximab was included in the chemotherapy regimen.

**Abstract:**

**Background:** The effect of HIV infection on the clinicopathological characteristics of diffuse large B-cell lymphoma (DLBCL) remains debatable. **Methods:** Fifty-three HIV-infected and ninety-three HIV-uninfected DLBCL patients were enrolled in the retrospective study by propensity score matching for sex, age, body mass index and international prognostic index (IPI) at a ratio of 1:2. The clinicopathological characteristics were compared between the two groups. **Results:** HIV-infected DLBCL patients had lower white blood cell counts [×10^9^/L; 4.4 (3.4–5.6) vs. 6.1 (4.2–8.2), *p* < 0.001], platelet counts (×10^9^/L; 184.7 ± 89.3 vs. 230.0 ± 113.9, *p* = 0.014) and serum albumin (g/L; 37.3 ± 6.9 vs. 41.3 ± 6.2, *p* < 0.001) but higher incidences of central nervous system (CNS) involvement (9.4% vs. 1.1%, *p* = 0.014), bone marrow involvement (24.5% vs. 11.5%, *p* = 0.044) and Epstein–Barr viremia (61.1% vs. 26.7%, *p* = 0.002) than HIV-uninfected patients. In terms of histopathology, HIV-infected patients had higher positivity of Epstein–Barr virus-encoded small RNA (EBER) (41.7% vs. 6.7%, *p* = 0.002), but lower CD20 (90.2% vs. 98.7%, *p*= 0.029) and CD79a (23.1% vs. 53.7%, *p* < 0.001) expression. The overall response rate (ORR) at the end of chemotherapy (70.2% vs. 87.8%, *p*= 0.012) and 1-year overall survival (OS) (61.7% vs. 84.2%, log-rank *p* = 0.006) in HIV-infected patients were significantly lower than those in HIV-uninfected patients. Multivariate analysis suggested IPI ≤2.0 [adjusted odds ratio (AOR) (95% confidence interval): 5.0 (1.2–21.2), *p* = 0.030] was associated with ORR, hypoalbuminemia [AOR: 3.3 (1.3–9.1), *p* = 0.018] and CNS involvement [AOR: 3.3 (1.0–10.5), *p* = 0.044] were associated with reduced 1-year OS in HIV-infected patients. **Conclusion:** HIV-infected DLBCL patients have unique blood profiles and phenotypic markers. Low ORR and 1-year OS were observed in HIV-infected DLBCL patients in our study, even in the HAART era.

## 1. Introduction

Human immunodeficiency virus (HIV) is associated with an increased incidence of malignances [1]. In particular, HIV-related non-Hodgkin lymphoma (NHL), including diffuse large B-cell lymphoma (DLBCL), is the most common hematological malignancy [2]. Although the incidence of NHL was significantly decreased in the highly active antiretroviral treatment (HAART) era, HIV-infected patients were at about 500-fold risk of NHL compared to HIV-uninfected patients [3,4]. Importantly, DLBCL is the most common subtype, accounting for 35–60% of all cases of reported NHL [5,6].

Currently, HIV infection is thought to contribute to lymphomagenesis through two indirect mechanisms as follows: (a) induction of B-cell activation and chronic inflammation. Some HIV-1 structural and regulatory proteins, such as gp120, Tat and p17 [7,8,9], induce chronic inflammation and persistent immune cell activation, which are key contributors to the development of NHL in the HIV-infected population [10,11]; and (b) loss of immunoregulatory control of oncogenic herpesviruses. HIV infection is associated with a high prevalence of Epstein–Barr virus (EBV) infection [11,12]. EBV expresses some oncogenic proteins, such as LMP1 and EBNA2, which have tumorigenic functions [13]. EBV infection is detected in a substantial proportion of HIV-related DLBCL patients [14]. Furthermore, the consequent attenuation of tumor surveillance caused by EBV and the HIV-related inflammatory environment increases the risk of lymphoma occurrence [12,15].

The effect of HIV infection on the outcome of the DLBCL population remains controversial. Some studies suggested that HIV infection was associated with lower overall survival rate. Meanwhile, some studies indicated HIV infection was not associated with poor prognosis of DLBCL patients [16,17,18]. In addition, HIV infection may alter phenotypic markers of DLBCL. For example, EBV-DNA and EBV-related virus proteins are more frequently detected in HIV-infected NHL patients than in HIV-uninfected NHL patients [11,13]. Exploring the different phenotypic characteristics between HIV-infected and HIV-uninfected DLBCL patients is helpful to understand the effect of HIV infection on DLBCL and even the outcome of patients.

Currently, the data on HIV-infected Chinese DLBCL patients are uncomprehensive. Some studies illustrated well the basic clinical characteristics of HIV-related DLBCL based on a small Chinese population [19,20,21]. Thus, a further study is necessary to enrich the data of HIV-infected Chinese DLBCL patients. In an effort to clarify this, we conducted a study to better understand the role of HIV infection in DLBCL. In the present retrospective study, 53 HIV-infected and 93 HIV-uninfected patients were enrolled after propensity score matching for sex, age, body mass index (BMI) and international prognosis index (IPI) at a ratio of 1:2. We compared the differences in laboratory tests, molecular characteristics and 1-year outcomes between the two groups, aiming to outline the different clinical features of DLBCL between HIV-infected and HIV-uninfected Chinese patients.

## 2. Methods

### 2.1. Study Design and Patient Selection

**Figure 1:**
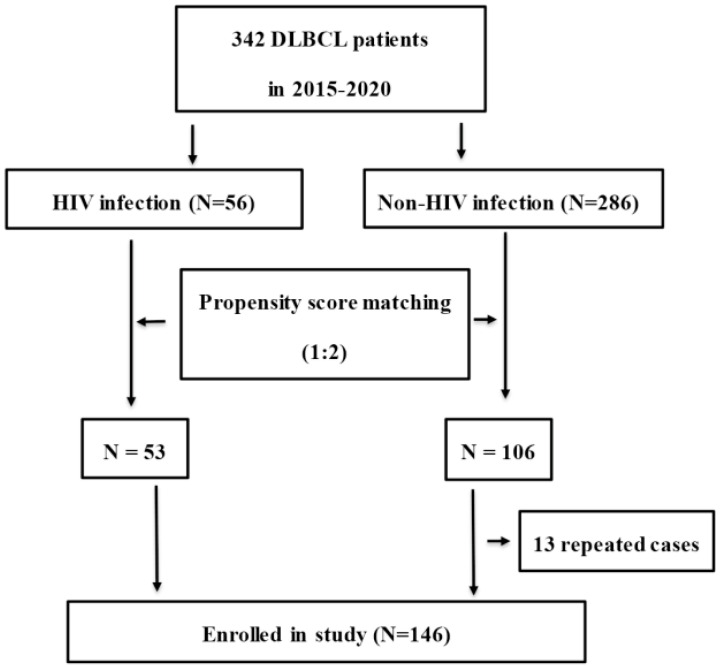
Study flowchart for patient selection. (DLBCL: diffuse large B-cell lymphoma).

Between January 2015 and December 2020, 342 (56 HIV-infected and 286 HIV-uninfected) DBBCL patients from the First Affiliated Hospital, College of Medicine, Zhejiang University, China, were eligible for enrollment in this retrospective study. Patients were excluded if they had liver cirrhosis, congestive heart failure, chronic kidney disorders, chronic obstructive pulmonary disease (COPD), solid organ malignancies or NHL relapse. DLBCL was diagnosed by pathological biopsy based on the 2016 revision of the World Health Organization classification of lymphoid neoplasms [22]. Propensity score matching for age, sex, BMI and IPI was used to select patients according to a ratio of 1:2 at a matching tolerance of 0.2. After 13 repeated cases were excluded, 53 HIV-infected and 93 HIV-uninfected patients were enrolled in the present study. The flowchart of patient selection is shown in Figure 1. Basic medical information of patients and chemotherapy regimens were recorded in the electronic medical records system (EMRS) of the hospital. Cases were followed up to December 2021 by clinical record, medical visiting and telephone interview.

### 2.2. Chemotherapy

The following chemotherapy regimens were included in the present study: CHOP ± R, EPOCH ± R and hyper CVAD/MA. The dosage and protocol of each chemotherapy are listed in Supplementary Table S1. Chemotherapy response was classified as complete response (CR), partial response (PR), stable disease (SD) or progressive disease (PD) according to the 2014 revised-response criteria for malignant lymphoma [23]. Overall response rate (ORR) was calculated as (the numbers of patients with CR + numbers of patients with PR)/total numbers of patients who accepted chemotherapy.

### 2.3. Statistical Analysis

Continuous normal variables are presented as the mean ± standard deviation, and continuous abnormal variables are presented as the median (interquartile range). Categorical variables are presented as the percentage (number). Student’s *t*-test or the Mann–Whitney *U* test was used to compare continuous variables. Chi-square or Fisher’s exact test was used to compare categorical variables. A receiver operating characteristic (ROC) curve was used to select the best threshold value. Logistic regression was used for the multivariate analysis of ORR. Patient survival was analyzed by the Kaplan–Meier method and Cox proportional hazards model. Lymphoma-related death was defined as an ‘event’. Factors with significance (*p* < 0.200) in univariate analysis were further analyzed in the multivariate Cox proportional hazards model or logistic regression by the forward stepwise (likelihood ratio) method. The odds ratio (OR) and 95% confidence interval (CI) were used to assess differences in survival between HIV-infected and HIV-uninfected groups. *p* < 0.050 was considered significant. All statistical data were analyzed using SPSS software 26.0 (SPSS Institute, Chicago, IL, USA) and GraphPad Prism 7.0 (GraphPad Software, La Jolla, CA, USA).

### 2.4. Ethics Approval

The research protocols were in accordance with the 1975 Declaration of Helsinki and received ethical approval from the Ethics Committee of the First Affiliated Hospital, College of Medicine, Zhejiang University (No. 2021-139). All data were analyzed anonymously. The committee waived the requirement for written informed consent from the participants.

## 3. Results

### 3.1. Baseline Characteristics of Patients before and after Propensity Score Matching

After propensity score matching, HIV-infected patients had lower WBCs [×10^9^/L; 4.4 (3.4–5.6) vs. 6.1 (4.2–8.2), *p* < 0.001] and neutrophils [×10^9^/L; 2.6 (1.5–3.8) vs. 3.6 (2.4–5.9), *p* < 0.001] compared to HIV-uninfected patients. There was no difference in hemoglobin between the two groups (*p* = 0.314). The platelet numbers in HIV-infected patients were also lower than those in HIV-uninfected patients [×10^9^/L; 184.7 ± 89.3 vs. 230.0 ± 113.9, *p* = 0.014]. Moreover, the albumin levels in HIV-uninfected patients (41.3 ± 6.2 g/L) were significantly higher than the albumin levels in HIV-infected patients (37.3 ± 6.9 g/L) (*p* < 0.001). There were no significant differences in the levels of alanine aminotransferase (ALT) (*p* = 0.382) and creatinine (*p* = 0.643) between the two groups. The serum urea level in HIV-infected patients was lower than that in HIV-uninfected patients [mmol/L; 4.5 (3.5–5.4) vs. 5.6 (4.6–6.9), *p* = 0.005).

Of the available data for 81 patients, 61.1% (22/36) HIV-infected patients and 26.7% (12/45) HIV-uninfected patients were with Epstein–Barr viremia (*p* = 0.002). Furthermore, the level of Epstein–Barr DNA was 4.3 ± 1.1 copies/mL (log_10_) in HIV-infected patients and 2.9 ± 1.3 copies/mL (log_10_) in HIV-infected patients (*p* < 0.001).

HIV-infected patients had higher incidences of CNS involvement (9.4% vs. 1.1%, *p* = 0.014), digestive involvement (54.7% vs. 18.3%, *p* < 0.001) and bone marrow involvement [24.5% vs. 11.5%, *p* = 0.044] compared to HIV-uninfected patients. The detailed characteristics and demographic information before and after propensity score matching were listed in Table 1.

### 3.2. Phenotypic Markers and Histopathology of HIV-Infected and HIV-Uninfected Patients

In the present study, phenotypic markers (CD3, CD5, CD10, CD20, CD21, CD56, CD79a, CD30, Bcl-2, Bcl-6, ALK, MUM1, Ki-67, PAX-5, CyclinD1 and MYC) were assayed on histopathology (Table 2). EBER was assayed by in situ hybridization. Of these markers, HIV-infected DLBCL patients had a high expression rate of EBER (41.7% vs. 6.7%, *p* = 0.002) but low expression rates of CD20 (90.2% vs. 98.7%, *p* = 0.029) and CD79a (23.1% vs. 53.7%, *p* < 0.001). No obvious differences in the frequencies of other molecular marks were found. Additionally, CD10, Bcl-6 and MUM1 expression were used to divide NHL into germinal center B-cell-like (GCB) and non-germinal center B-cell-like (non-GCB) subgroups according to Hans algorithm. Among the DLBCL patients, 44.7% (21/47) of HIV-infected patients and 34.2% (26/76) of HIV-uninfected patients were considered GCB (*p* = 0.208).

### 3.3. Chemotherapy Associated ORR between HIV-Infected and HIV-Uninfected DLBCL Patients

Of 146 DLBCL patients, 137 (93.8%) patients accepted chemotherapy, and 9 (6.2%) patients did not accept chemotherapy. Notably, 6 (11.3%) of HIV-infected patients and 3 (3.2%) of HIV-uninfected patients abandoned treatment (*p* = 0.073).

Of 137 patients who accepted chemotherapy, 103 (75.2%) patients were treated with the CHOP ± R regimen, 9 (6.6%) patients were treated with the EOPCH ± R regimen, 2 (1.5%) patients were treated with the hyper CVAD/MA regimen, and 23 (16.8%) patients were treated with other chemotherapy regimens. Moreover, 112 (81.8%) patients achieved ORR (91 patients achieved CR and 21 patients achieved PR), but 25 (18.2%) patients did not benefit from treatments at the end of 6–8 cycles of chemotherapy. The ORR was close between the CHOP regimen and the non-CHOP regimen among patients who accepted chemotherapy (*p* = 0.152). The ORR was 70.2% (33/47) in HIV-infected patients and 87.8% (79/90) in HIV-uninfected patients (*p* = 0.012), and the ORR was 88.3% (83/94) in patients who were treated with rituximab and 59.6% (31/52) in patients who were treated without rituximab at the end of chemotherapy (*p* < 0.001).

Factors associated with ORR at the end of chemotherapy were analyzed in patients who accepted chemotherapy. In univariate analysis, serum albumin ≥35.0 g/L [OR: 3.0 (1.1–8.1), *p* = 0.026] and rituximab usage [OR: 3.6 (1.5–8.9), *p* = 0.003] were positively related to ORR, whereas HIV infection [OR: 0.3 (0.1–0.8), *p* = 0.012], CNS involvement [OR: 0.1 (0.0–0.8), *p* = 0.042] and gastrointestinal involvement [OR: 0.4 (0.2–1.0), *p* = 0.048] were negatively associated with ORR. In addition, BMI >21.0 kg/m^2^ [OR: 1.8 (0.8–4.5), *p* = 0.158] and IPI ≤ 2.0 [OR: 3.0 (1.0–1.4), *p* = 0.063] were marginally associated with ORR. Multivariate logistic regression analysis indicated that rituximab usage [AOR: 4.0 (1.5–10.2), *p* = 0.004], no gastrointestinal involvement [AOR: 2.7 (1.1–7.1), *p* = 0.039] and no CNS involvement [AOR: 9.2 (1.3–65.1), *p* = 0.027] were independent prognostic factors for ORR. HIV infection was not an independent risk for decreased ORR (*p* = 0.784) (Table 3).

Furthermore, the factors associated with ORR in HIV-infected patients were analyzed. We found that IPI ≤ 2.0 [OR: 1.9 (1.1–3.0), *p* = 0.023], no marrow involvement [OR: 2.2 (0.8–4.9), *p* = 0.163] and rituximab usage [OR: 2.4 (0.7–8.9), *p* = 0.170] were factors associated with ORR in univariate model, while multivariate logistic regression analysis indicated that only an IPI ≤ 2.0 [OR: 5.0 (1.2–21.2), *p* = 0.030] was related to the effectiveness of chemotherapy (Table 3).

### 3.4. One-Year Overall Survival (OS) of HIV-Infected and HIV-Uninfected DLBCL Patients

**Figure 2:**
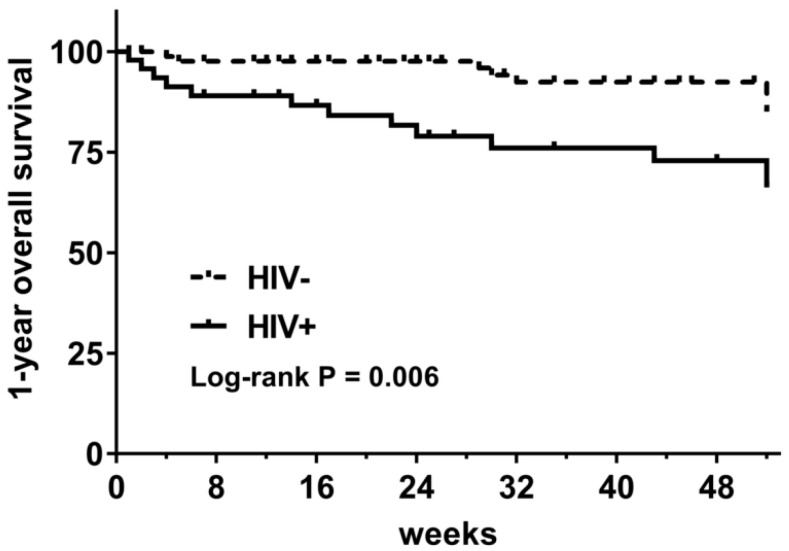
One-year overall survival of DLBCL patients with and without HIV infection. (HIV−: HIV-uninfected patients; HIV+: HIV-infected patients).

Among 137 DLBCL patients who accepted chemotherapy, 15.3% (21/137) died during the follow-up period, including 27.7% (13/47) of HIV-infected patients and 8.9% (8/90) of HIV-uninfected patients (*p* = 0.004). The 1-year OS was 66.2% in HIV-infected patients and 85.1% in HIV-uninfected patients (log-rank *p* = 0.006) (Figure 2).

The factors associated with 1-year mortality of patients who accepted chemotherapy were analyzed. In the univariate Cox proportional hazard model, HIV infection [OR: 3.2 (1.3–7.6), *p* = 0.010], CNS invasion [OR: 3.9 (2.1–13.2), *p* = 0.030], chemotherapy without rituximab [HR: 4.7 (1.9–11.5), *p* = 0.001], serum albumin < 35.0 g/L [OR: 4.7 (2.0–11.2), *p* < 0.001], hemoglobin < 110.0 g/L [OR: 2.0 (0.8–4.7), *p* = 0.120] and IPI > 2.0 [OR: 4.0 (1.0–5.7), *p* = 0.054] were closely associated with 1-year mortality. In the multivariate Cox proportional hazard model, we found that CNS invasion [AOR: 3.8 (1.1–13.8), *p* = 0.039], serum albumin < 35.0 g/L [AOR: 4.4 (1.8–10.9), *p* = 0.001] and chemotherapy without rituximab [AOR: 3.8 (1.5–9.4), *p* = 0.005] were independent risk factors for the 1-year mortality rate (Table 4).

Furthermore, survival analysis was performed in HIV-infected patients who received chemotherapy. In the univariate model, we found that only serum albumin ≥ 35.0 g/L [OR: 4.6 (1.5–13.7), *p* = 0.007] was associated with 1-year OS. In addition, hemoglobin < 110.0 g/L [OR: 2.5 (0.8–7.5), *p* = 0.101], CNS invasion [OR: 3.1 (0.9–11.3), *p* = 0.086] and IPI > 2.0 [OR: 2.6 (0.8–8.5), *p* = 0.111] were marginally related to 1-year mortality. In the multivariate model, CNS invasion [OR: 3.3 (1.0–10.5), *p* = 0.044] and serum <35.0 g/L [OR: 3.3 (1.3–9.1), *p* = 0.018] were independent risk factors for the 1-year mortality rate (Table 4).

In addition, among 43 HIV-infected DBLCL patients who accepted chemotherapy and were without CNS invasion, we found that the 1-year mortality was 9.1% (2/22) in those treated with rituximab and 38.1% (8/21) in those treated without rituximab [OR: 6.2 (1.1–33.7), *p* = 0.024], and the 1-year mortality was 50.0% (6/12) in patients with serum albumin < 35.0 g/L and 9.3% (4/31) in patients with serum albumin ≥35.0 g/L [OR: 6.8 (1.4–31.6), *p* = 0.010]. No difference in mortality was found between patients with different levels of hemoglobin (g/L; < 110.0 vs. ≥ 110.0, *p* = 0.131), IPI (≤ 2.0 vs. > 2.0, *p* = 0.174) and BMI (kg/m^2^; <21.0 vs. ≥ 21.0, *p* = 0.440).

## 4. Discussion

As early as the initial stage of the HIV/AIDS epidemic, the high incidence of AIDS-related malignances, including Kaposi sarcoma and non-Hodgkin’s lymphoma, were predominant hematological malignancies in HIV-infected patients. With the increasing accessibility of antiviral therapy and improving medical care for HIV/AIDS, AIDS-related cancers have dramatically decreased. Meanwhile, the incidence of non-AIDS-related cancers rise in HIV-infected patients in Western countries [24,25]. More recent data from studies of HIV-infected people in the US, Europe and Australia indicated that non-AIDS-related cancers increased, reflecting increasing rates in the general population [26,27]. In developing countries, including China, there are a large proportion of newly-HIV infected people, AIDS-related cancers are the most common malignances [24,28].

To date, the differences in the clinical and molecular characteristics of HIV-infected and HIV-uninfected DLBCL patients are controversial. The present retrospective study had the following findings: (1) in general, HIV-infected patients were predominantly male, younger and had a lower BMI; (2) after propensity score matching for age, sex, BMI and IPI at a ratio of 1:2 with HIV-uninfected patients, HIV-infected patients had a higher incidence of leukopenia, thrombocytopenia, hypoalbuminemia and Epstein–Barr viremia; (3) the EBER expression rate was high in HIV-infected patients, but the CD20 and CD79a expression rates were low on histopathology in HIV-infected patients; (4) ORR at end of chemotherapy and 1-year OS in HIV-infected DLBCL patients who accepted chemotherapy were lower in the univariate model, though those results were not confirmed in the multivariate model; (5) high IPI was related to decreased ORR. Meanwhile, hypoalbuminemia and CNS involvement were independent risk factors for decreased 1-year OS in HIV-infected DLBCL patients.

In general, HIV-infected NHL patients had lower BMI and were younger than HIV-uninfected patients. Even after propensity score matching, HIV-infected patients had high incidence of leukopenia, thrombocytopenia, hypoalbuminemia, CNS invasion, bone marrow involvement and Epstein–Barr viremia.

Our data showed that HIV-infected patients displayed high EBER expression but low CD20 and CD79a expression according to histopathological analysis. It seemed that HIV-associated lymphoma frequently occurs at a much higher frequency in EBV-DNA positive patients than in EBV-DNA negative patients [29,30]. CD20 and CD79a are the most widely used B-cell markers [31]. According to histopathological analysis, our data showed that the positivity of CD20 (90.2%) and CD79a (23.1%) was lower in HIV-infected patients. It was reported that 2–26% of HIV-infected DLBCL patients were negative for CD20 [32,33]. The exact mechanisms for loss of CD20 and CD79a expression were not clearly defined. Some factors associated with the loss of CD20 were inflection of receptor, bias of laboratory test and differences on biopsy site [34]. However, it was also suggested that CD20-negative patients presented with more extranodal disease, bone involvement, conversion of low-grade lymphoma to high-grade lymphoma, and even inferior survival rate [34,35]. Clinicopathological data showed that CD79a-negative expression was observed in patients with decreased platelet numbers or with high-grade lymphoma [36]. Our data indicated that HIV-infected DLBCL patients had high incidence of bone involvement and thrombocytopenia, which might relate to low expression of CD20 and CD79a on histopathology.

Although HIV infection was not a factor for decreased ORR and 1-year OS in multivariate analysis, our data indicated that both ORR and 1-year OS of HIV-infected patients were generally lower than in HIV-uninfected patients. The ORR in the present study was close to the ORR previously reported in other studies in China [19]. IPI > 2.0 was the only factor associated with decreased ORR in the multivariate analysis. In addition, the 1-year OS (66.2%) in HIV-infected patients was also close to the data reported in other studies in the Chinese population [19,21]. Importantly, our data demonstrated that hypoalbuminemia (<35.0 g/L) and CNS involvement were independent risk factors for reduced 1-year OS in HIV-infected BLDCL patients. Some studies indicated that hypoalbuminemia was associated with increased mortality in patients with lymphoma [37,38]. Consistent with other studies [39,40], our data also suggested that CNS involvement was an independent risk factor for poor OS in DLBCL patients. Therefore, it is rational that hypoalbuminemia and CNS invasion were linked to low 1-year OS in HIV-infected DLBCL patients.

There were some other potential factors related to the high mortality of HIV-infected DLBCL patients. We found that HIV-infected DLBCL patients had a higher incidence of leukopenia, neutropenia, thrombocytopenia, hypoalbuminemia, bone marrow involvement and Epstein–Barr viremia. These hematological abnormalities are mainly related to decreased production of precursor cells and increased destruction of peripheral differentiated cells resulting from a higher incidence of lymphoma invasion into the bone marrow in HIV-infected patients. Previous studies have shown that hypoalbuminemia, leukopenia and Epstein–Barr viremia were associated with a worse outcome of HIV-infected DLBCL patients [41,42].

Considering the substantial overweight of CNS involvement on mortality in multivariate analysis, we revaluated the factors associated with 1-year OS in HIV-infected patients without CNS involvement. Our study demonstrated that the odds ratio of mortality was 6.2-fold for patients who accepted chemotherapy without rituximab and 6.8-fold for patients with hypoalbuminemia. These data implied that administration of rituximab intravenously may effectively improve the outcome of BLBCL patients without CNS involvement.

The present study had several limitations. First, the present study indicated that HIV-infected patients had different phenotypic markers from HIV-uninfected patients. For example, histopathological analysis indicated a high positive rate of EBER. However, we did not discuss the effect of some phenotypic markers (such as Bcl-2 and c-Myc) on patient outcomes due to inadequate frequencies of those markers. Second, the present study was based on a small number of cases and a short observation period. Some potential risk factors (such as HIV-RNA load) were not analyzed, which may have produced potential effects on our results and conclusions. Third, we only provided 1-year OS rather than progression-free survival of patients. Thus, a further study based on large population is necessary for clarifying the detailed causes of mortality in HIV-infected DLBCL patients.

## 5. Conclusions

In conclusion, we found that HIV-infected DLBCL patients had different blood and histopathological profiles from HIV-uninfected patients. HIV-infected DLBCL patients had low ORR and 1-year OS, which may be associated with the higher incidence of leukopenia, neutropenia, thrombocytopenia and hypoalbuminemia as well as high involvement of the CNS, gastrointestinal tract and bone marrow. A high IPI was associated with poor ORR, and hypoalbuminemia and CNS involvement were independent risk factors for 1-year OS. HIV-infected DLBCL patients without CNS involvement had a favorable outcome if rituximab was included in the chemotherapy regimen.

## Figures and Tables

**Table 1:** The demographic characteristics of NHL patients with and without HIV infection.

Parameter	Before PSM		*p* Value	After PSM		*p* Value
	HIV− (*n* = 286)	HIV+ (*n* = 56)		HIV− (*n* = 93)	HIV+ (*n* = 53)	
**Age (years)**	56.6 ± 13.9	48.8 ± 14.5	<0.001	52.5 ± 14.1	50.1 ± 13.9	0.316
**Sex [%(*n*)]**						
Male	55.9 (160/286)	85.7 (48/56)	<0.001	82.8 (77/93)	84.9 (45/53)	0.741
**Body mass index**	22.8 ± 3.4	21.8 ± 3.1	0.046	22.2 ± 2.9	22.2 ± 2.8	0.963
**GCB**	35.5 (77/217)	46.9 (23/49)	0.135	34.2 (26/76)	44.7 (21/47)	0.208
**IPI**	2.0 (1.0–3.0)	2.0 (1.0–3.0)	0.107	2.0 (1.0–3.0)	2.0 (1.0–3.0)	0.308
**Arbor score**	3.0 (2.0–4.0)	3.0 (2.0–4.0)	0.548	4.0 (2.0–4.0)	3.0 (1.0–4.0)	0.694
**ECOG score**	1.0 (0.0–2.0)	1.0 (0–3.0)	0.012	1.0 (0.0–2.0)	2.0 (1.0–2.8)	<0.001
**CNS involvement [%(*n*)]**	1.9 (2/106)	12.5 (7/56)	0.005	1.1 (1/93)	9.4 (5/53)	0.014
**GI involvement [%(*n*)]**	44.9 (48/107)	55.4 (31/56)	0.203	18.3 (17/93)	54.7 (29/53)	<0.001
**Marrow involvement [%(*n*)]**	9.3 (10/107)	23.2 (13/56)	0.016	11.5 (10/87)	24.5 (13/53)	0.044
**Pulmonary involvement [%(*n*)]**	6.6 (7/106)	12.5 (7/56)	0.204	5.0 (2/40)	11.3 (6/53)	0.282
**Blood test**						
WBC (×10^9^/L)	5.6 (4.4–7.7)	4.3 (3.2–5.6)	<0.001	6.1 (4.2–8.2)	4.4 (3.4–5.6)	<0.001
Neutrophils (×10^9^/L)	3.6 (2.5–5.6)	2.9 (1.9–4.0)	0.001	3.6 (2.4–5.9)	2.6 (1.5–3.8)	<0.001
Hemoglobin (g/L)	120.8 ± 23.7	114.4 ± 23.7	0.068	120.5 ± 28.8	115.8 ± 22.9	0.314
platelet (×10^9^/L)	227.1 ± 104.4	182.3 ± 91.0	0.003	230.0 ± 113.9	184.7 ± 89.3	0.014
**Biochemical profile**						
Abumin (g/L)	40.6 ± 7.0	37.2 ± 6.7	0.001	41.3 ± 6.2	37.3 ± 6.9	<0.001
ALT (U/L)	18.0 (11.0–27.0)	19.0 (12.0–26.0)	0.369	22.0 (12.0–31.7)	18.0 (12.0–25.0)	0.382
LDH (U/L)	226.0 (181.5–344.0)	289.0 (190.0–465.0)	0.064	229.5 (185.0–345.0)	291.0 (197.0–477.5)	0.141
Creatinine (μmol/L)	65.0 (56.0–78.0)	71.0 (56.0–85.0)	0.376	70.0 (59.5–84.0)	71.0 (57.0–85.0)	0.643
Urea (mmol/L) eGFR (mL/min)	5.0 (4.1–6.4) 100.2 ± 28.5	4.4 (3.4–5.4) 113.7 ± 42.3	0.061 0.006	5.6 (4.6–6.9) 100.3 ± 29.7	4.5 (3.5–5.4) 110.5 ± 41.3	0.005 0.102

HIV−: HIV-uninfected patients; HIV+: HIV-infected patients; GCB: germinal center B-cell-like lymphoma; IPI: international prognostic index; ECOG: Eastern Collaborative Oncology Group; CNS: central nervous system; GI: gastrointestinal; WBC: white blood cells; ALT: alanine aminotransferase; LDH: lactate dehydrogenase; eGFR: estimated glomerular filtration rate.

**Table 2:** Comparison of phenotypic markers in DLBCL patients with and without HIV infection.

Markers	HIV− Patients (*n* = 93)	HIV+ Patients (*n* = 53)	*p*-Value
CD3	14.9 (10/67)	25.6 (10/39)	0.174
CD5	9.3 (5/54)	19.4 (6/31)	0.198
CD10	28.6 (18/63)	43.2 (16/37)	0.135
CD20	98.7 (77/78)	90.2 (37/41)	0.029
CD21	34.6 (9/26)	21.7 (5/23)	0.319
CD56	7.1 (1/14)	11.1 (1/9)	-
CD79a	53.7 (50/93)	23.1 (12/52)	<0.001
CD30	21.9 (7/32)	43.8 (7/16)	0.178
EBER	6.7 (2/30)	41.7 (10/24)	0.002
Bcl-2	46.3 (38/82)	45.3 (24/53)	0.904
Bcl-6	85.5 (53/62)	85.3 (29/34)	0.980
ALK	0.0 (0/22)	0.0 (0/16)	-
MUM1	78.3 (47/60)	72.7 (24/33)	0.543
Ki-67 ≥85.5%	30.0 (21/70)	36.1 (13/36)	0.532
<85.5%	70.0 (49/70)	63.9 (23/36)	
PAX-5	90.6 (29/32)	100.0 (18/18)	0.544
CyclinD1	0 (0/27)	6.3 (1/16)	-
c-Myc (>40%)	68.8 (13/19)	58.8 (10/17)	0.549
Myc/Bcl-2 DPE+	10.8 (10/93)	11.3 (6/53)	0.916

HIV−: HIV-uninfected patients; HIV+: HIV-infected patients; cMYC staining of >40% of neoplastic cells together with BCL2 expression >70% was interpreted as positive staining and defined the “double-protein expressers” (DPE).

**Table 3:** Risk factors for overall response rate in DLBCL patients who accepted chemotherapy.

Factor	All Patients (*n* = 137)					HIV+ Patients (*n* = 47)				
	ORR (*n* = 112)	Univariate		Multivariate		ORR (*n* = 33)	Univariate		Multivariate	
		OR (95% CI)	*p*	AOR (95% CI)	*p*		OR (95% CI)	*p*	AOR (95% CI)	*p*
**Sex**			0.473							
Male	96/116	1.1 (0.8–1.4)			-	29/40	0.5 (0.1–2.6)	0.412		-
Female	5/16	1				4/7	1			
**Age (years)**			0.174							
<50.0	46/60	1.8 (0.8–4.4)			0.421	17/23	1	0.587		-
≥50.0	66/77	1				16/24	0.7 (0.2–2.5)			
**BMI (kg/m^2^)**										
<21.0	37/49	1	0.158		0.094	11/18	1	0.282		
≥21.0	75/88	1.8 (0.8–4.5)				22/29	2.0 (0.6–7.1)			
**Serum albumin (g/L)**										
Missing data	1/1	-	0.026		0.199		-			
<35	15/23	1				8/13	1	0.421		
≥35	96/113	3.0 (1.1–8.1)				25/34	1.7 (0.4–6.7)			
**rituximab**			0.003							
on	83/94	3.6 (1.5–8.9)		4.0 (1.5–10.2)	0.004	14/23	2.4 (0.7–8.9)	0.170		0.133
off	29/43	1		1		19/24	1			
**Hemoglobin (g/L)**										
Missing data	1/1									
<110.0	34/44	1	0.366		0.508	11/18	2.0 (0.6–7.1)	0.282		
≥110.0	77/92	1.5 (0.6–3.7)				22/29	1			
**HIV infection**			0.012		0.784					
No	79/90	1								
Yes	33/47	0.3 (0.1–0.8)								
**CNS involvement**										
Yes	2/5	0.1 (0.0–0.8)	0.042	0.1 (0–0.8)	0.027	2/4	1	0.572		
No	110/132	1		1		31/43	0.4 (0.0–3.0)			
**GI involvement**										
Yes	31/43	0.4 (0.2–1.0)	0.048	0.4 (0.1–1.0)	0.039	18/27	0.7 (0.2–2.4)	0.537		
No	81/94	1		1		15/20	1			
**Bone marrow involvement**										
Missing data	6/6									
Yes	17/23	1.5 (0.7–3.4)	0.383			7/13	0.4 (0.1–1.4)	0.163		0.266
No	89/108	1				26/34	1			
**IPI score**			0.063							
Missing data	1/1							0.023		0.030
≤2.0	67/77	1.2 (1.0–1.4)			0.204	19/22	1.9 (1.1–3.0)		5.0 (1.2–21.2)	
>2.0	44/59	1				14/25	1		1	
**LDH (U/L)**			0.097							
Missing data	10/12					10/18				
<250.0	55/63	1			0.189	23/31	1	0.876		
≥250.0	47/62	0.5 (0.2–1.2)				21/29	0.9 (0.3–2.9)			
**CD4 (cells/mm^3^)**										
Missing data	80/91					1/1				
<100.0	12/17					12/17	1			
≥100.0	20/20					20/29	0.9 (0.3–3.4)	0.908		
**ART**										
On						18/26	1			
Off						15/21	0.9 (0.3–3.2)	0.870		

OR: Odds ratio; ORR: overall response rate; HIV+ patients: HIV-infected patients; BMI: body mass index; HIV: human immunodeficiency virus; CNS: central nervous system; GI: gastrointestinal; IPI: international prognostic index; LDH: lactate dehydrogenase; ART: antiviral therapy.

**Table 4:** Risk factors for 1-year mortality in DLBCL patients in univariate/multivariate Cox proportional hazards models.

Factor	All Patients (*n* = 137)					HIV+ Patients (*n* = 47)				
	1-Year Mortality (*n* = 21)	Univariate		Multivariate		1-Year Mortality (*n* = 13)	Univariate		Multivariate	
		OR (95% CI)	*p*	AOR (95% CI)	*p*		OR (95% CI)	*p*	AOR (95% CI)	*p*
**Sex**			0.182							
Male	16/116	2.0 (0.7–5.4)				10/40	0.5 (0.1–2.0)	0.541		
Female	5/21	1				3/7	1			
**Age (years)**			0.709							
<60.0	15/90	1				9/34	1	0.533		
≥60.0	6/47	0.8 (0.3–2.2)				4/13	1.5 (0.4–4.7)			
**BMI (kg/m^2^)**										
<21.0	9/49	1.6 (0.7–3.8)	0.299			6/18	0.6 (0.2–1.8)	0.591		
≥21.0	12/88	1				7/29	1			
**Serum albumin (g/L)**										
Missing data	0/1		<0.001		0.001	0				0.018
<35	9/23	4.7 (2.0–11.2)		4.4 (1.8–10.9)		7/13	4.6 (1.5–13.7)	0.007	3.3 (1.3–9.1)	
≥35	12/113	1		1		6/34	1		1	
**rituximab**			0.001							
on	7/94	1		1	0.005	4/24	1	0.214		0.060
off	14/43	4.7 (1.9–11.5)		3.8 (1.5–9.4)		9/23	2.1 (0.7–6.9)			
**Hemoglobin (g/L)**										
Missing data	0/1									
<110.0	9/44	2.0 (0.8–4.7)	0.120		0.570	7/18	2.5 (0.8–7.5)	0.101		0.075
≥110.0	12/92	1				6/29	1			
**HIV infection**			0.010		0.374					
No	8/90	1								
Yes	13/47	3.2 (1.3–7.6)								
**CNS involvement**										
Yes	3/5	3.9 (1.1–13.2)	0.030	3.8 (1.1–13.8)	0.039	3/4	1	0.086	1	0.044
No	18/132	1		1		10/43	3.1 (0.9–11.3)		3.3 (1.0–10.5)	
**GI involvement**										
**Yes**	9/43	2.0 (0.8–4.7)	0.127		0.144	8/27	1.7 (0.6–5.2)	0.352		
**No**	12/94	1				5/20	1			
**Bone marrow involvement**										
**Yes**	5/23	1.5 (0.5–4.1)	0.433			5/13	1.8 (0.6–5.4)	0.318		
**No**	16/108	1				8/34	1			
**IPI score**			0.054							
Missing data	0/1					0				
≤2.0	8/77	1			0.702	4/22	1	0.111		0.797
>2.0	13/59	4.0 (1.0–5.7)				9/25	2.6 (0.8–8.5)			
**CD4 (cells/mm^3^)**										
Missing data	9/91					1				
<100.0	4/17		-			4/17	0.8 (0.2–2.6)	0.692		
≥100.0	8/29	-				8/29	1			
**ART**										
on						8/26	1			
off						5/21	1.4 (0.4–5.2)	0.596		

OR: Odds ratio; AOR: adjusted odds ratio; HIV + patients: HIV-infected patients; BMI: body mass index; HIV: human immunodeficiency virus; CNS: central nervous system; GI: gastrointestinal; IPI: international prognostic index; LDH: lactate dehydrogenase; ART: antiviral therapy.

## Data Availability

The data that support the findings of this study are available from the corresponding author upon reasonable request.

## Supplementary Materials

The following supporting information can be downloaded at: https://www.mdpi.com/article/10.3390/cancers14215380/s1, Table S1: The dosage and protocol for each chemotherapy regimen.
