# Supplementary material for: Clinical Features, Phenotypic Markers and Outcomes of Diffuse Large B-Cell Lymphoma between HIV-Infected and HIV-Uninfected Chinese Patients
Source: Cancers (Basel). 2022 Oct 31;14(21):5380. doi: 10.3390/cancers14215380 (PMC9655449; doi:10.3390/cancers14215380)
Supplement: Supplementary file 1 [file cancers-14-05380-s001.zip › cancers-1909305-supplementary.pdf]

Table S1. The dosage and protocol for each chemotherapy regimen.

Components	Dosage and protocol	days
**CHOP ± R**		
Cyclophosphamide (IV)	750 mg/m2	1
Vincristine (IV)	1.4 mg/m2	1
Doxorubicin (IV)	50 mg/m2	1
Prednisolone (PO)	60 mg/m2	1-5
Rituximab* (IV)	375 mg/m2	0
**EPOCH ± R**		
Etoposide (IV)	50 mg/m2 for 96 hours	1-4
Vincristine (IV)	0.4 mg/m2 for 96 hours	1-4
Doxorubicin (IV)	12 mg/m2 for 96 hours	1-4
Cyclophosphamide (IV)	750 mg/m2	5
Prednisolone (PO)	60 mg/m2	1-5
Rituximab* (IV)	375 mg/m2	0
**Hyper-CADV/MA**		
Hyper-CVAD/induction		
Cyclophosphamide (IV)	300 mg/m2 per 12h	1-3
Vincristine (IV)	2 mg	4, 11
Doxorubicin (IV)	50 mg/m2	4
Dexamethasone (PO or IV)	40 mg	1-4, 11-14
MA/consolidation		
Methotrexate (IV)	200 mg/m2 over 2h then 800 mg/m2 over 22h	1
Cytarabine (IV)	3 g/m2 per 12h	2, 3

* Rituximab was a component of CHOP-R and EPOCH-R regemin.
